# A Novel Epigenetic Machine Learning Model to Define Risk of Progression for Hepatocellular Carcinoma Patients

**DOI:** 10.3390/ijms22031075

**Published:** 2021-01-22

**Authors:** Luca Bedon, Michele Dal Bo, Monica Mossenta, Davide Busato, Giuseppe Toffoli, Maurizio Polano

**Affiliations:** 1 Experimental and Clinical Pharmacology Unit, Centro di Riferimento Oncologico di Aviano (CRO), IRCCS, 33081 Aviano (PN), Italy; luca.bedon@cro.it (L.B.); mdalbo@cro.it (M.D.B.); monica.mossenta@cro.it (M.M.); davide.busato@cro.it (D.B.); 2 Department of Chemical and Pharmaceutical Sciences, University of Trieste, Via L. Giorgieri 1, 34127 Trieste, Italy; 3 Department of Life Sciences, University of Trieste, 34127 Trieste, Italy

**Keywords:** hepatocellular carcinoma, epigenetic, prediction model, tumor microenvironment, hepatocellular carcinoma DNA methylation

## Abstract

Although extensive advancements have been made in treatment against hepatocellular carcinoma (HCC), the prognosis of HCC patients remains unsatisfied. It is now clearly established that extensive epigenetic changes act as a driver in human tumors. This study exploits HCC epigenetic deregulation to define a novel prognostic model for monitoring the progression of HCC. We analyzed the genome-wide DNA methylation profile of 374 primary tumor specimens using the Illumina 450 K array data from The Cancer Genome Atlas. We initially used a novel combination of Machine Learning algorithms (Recursive Features Selection, Boruta) to capture early tumor progression features. The subsets of probes obtained were used to train and validate Random Forest models to predict a Progression Free Survival greater or less than 6 months. The model based on 34 epigenetic probes showed the best performance, scoring 0.80 accuracy and 0.51 Matthews Correlation Coefficient on testset. Then, we generated and validated a progression signature based on 4 methylation probes capable of stratifying HCC patients at high and low risk of progression. Survival analysis showed that high risk patients are characterized by a poorer progression free survival compared to low risk patients. Moreover, decision curve analysis confirmed the strength of this predictive tool over conventional clinical parameters. Functional enrichment analysis highlighted that high risk patients differentiated themselves by the upregulation of proliferative pathways. Ultimately, we propose the oncogenic *MCM2* gene as a methylation-driven gene of which the representative epigenetic markers could serve both as predictive and prognostic markers. Briefly, our work provides several potential HCC progression epigenetic biomarkers as well as a new signature that may enhance patients surveillance and advances in personalized treatment.

## 1. Introduction

Hepatocellular carcinoma (HCC) is one of the leading causes of cancer deaths worldwide. According to the 2018 statistical report of global cancer burden (GLOBOCAN), HCC is the sixth for incidence and the fourth for mortality cancer, accounting for 841,080 new cases and 781,631 deaths per year worldwide [1]. HCC lesions originate from chronic liver fibrosis and cirrhosis, which arise from repeated cycles of injury and repair. Tissue injuries originate from several sources including chronic viral hepatitis (hepatitis B and C), excessive alcohol intake, non-alcoholic fatty liver disease, aflatoxin exposure, tobacco smoking and diabetes [2,3].

With its consistent immune/inflammatory pathogenesis, HCC appears as a strong candidate for the application of immune-based therapies; however, this strategy has been shown to be only partially successful [4]. Currently, the survival of HCC patients that are not eligible for curative therapy (i.e., resection, local ablation and liver transplantation) depends on their response to the less efficient systemic chemotherapy [5,6]. The refractoriness of HCC, not only to classical chemotherapy but also to targeted therapy, is still poorly understood; HCC progression and resistance can be affected by multiple biological processes such as epigenetic modulation [7], immune microenvironment in the tumor site [8] and mechanisms of chemoresistance (MOC) [9]. Therefore, HCC patient stratification into homogeneous progression groups is critical for the identification of potential biological processes involved in cancer progression, which then form the bases for the selection of the most appropriate treatment or possibly shed new light on novel druggable biological targets.

Despite the extensive advancement in earlier diagnosis, therapy decision-making and interdisciplinary evaluation, the prognosis of HCC patients remains poor. Ongoing prognostic models integrate tumor node metastasis (TNM) staging, liver function, comorbidities and other parameters to predict HCC progression and prognosis [10]; however, since HCC is a very heterogeneous disease, the prognostic performance of classical methods is still not satisfactory. Nowadays taking into account large scale omics data is becoming fundamental when establishing novel prognostic and predictive tools that can better represent a broader HCC scenario.

The use of whole gene expression and methylation analysis of tumors have proved that it is possible to highlight patterns and signatures related to prognosis, tumor classification and response to treatment [11,12]. Meanwhile, Machine Learning methods have been trained and applied into genomic data to discover new molecular signatures, interpret complex biological mechanisms and predict clinical outcomes from biomedical datasets [13,14]. Therefore, besides traditional clinical-pathological risk factor models, an efficient predictive model that can classify patients in different cancer progression groups is highly desirable. Moreover, the features used by this model for making predictions could be the bricks of an optimized prognostic model capable of stratifying patients in relation to cancer progression, eventually providing a more oriented therapy decision and an improved clinical management.

In this study, we aimed to build and evaluate a predictive model able to classify HCC patients with a progression-free survival (PFS) time greater or less than six months by using their methylation profiles. HCC patients were from a large dataset within the Liver Hepatocellular Carcinoma (LIHC) project of The Cancer Genome Atlas (TCGA). We initially used a Machine Learning algorithm (Random Forest) combined with different features selection algorithms to select the best prediction subset of methylation probes on cytosine-phosphate-guanine dinucleotides (CpGs), resulting in a final 34 CpGs-based model for PFS prediction. Then, starting from these final 34 markers, we performed a univariate Cox regression analysis to select PFS relevant CpG probes followed by the construction of a CpGs-based prognostic signature using a stepwise model selection. A four-CpGs-based risk model was successfully built, validated and used to stratify the patients in high risk and low risk for an early cancer progression. Finally, we conducted an analysis of differentially expressed genes (DEGs) followed by a functional enrichment analysis to gain more insights into the biological differences between high risk and low risk patients as well as into the processes involved. This prognostic signature could be useful in the HCC patient administration, by providing a stratification system that reliably separates patients with respect to the progression prognosis, ultimately impacting both therapy and clinical decision-making.

## 2. Materials and Methods

### 2.1. Datasets

The transcriptome data (HTSeq raw read counts), methylation data (beta values), survival information and clinicopathological information within the Liver Hepatocellular Carcinoma (LIHC) project of The Cancer Genome Atlas (TCGA) were downloaded from the GDC data repository (portal.gdc.cancer.gov).

The patients were divided in relation to the PFS time in two groups: a first “G6M” group characterized by a PFS greater than six months, and a second “L6M” group characterized by a PFS less than six months.

DNA methylation dataset based on the Illumina HumanMethylation450 BeadChip Assay (version 07-20-2019) includes the analysis of 374 primary tumor specimens. The genomic annotation of each CpG probe was accomplished using the illuminaMethyl450_ hg38_GDC manifest from GDG portal. The methylation level of each CpG was expressed as a ratio of intensities between methylated and unmethylated alleles ranging from 0 to 1 (β=M/(M+U)). The probes were filtered out based on the following criteria: first, methylation beta value not available in any sample; second, probes located in sex chromosomes [15]; third, highly correlated probes (Pearson r > |0.9|) [16]; fourth, probes containing single-nucleotide polymorphisms (dbSNP132Common) [17]; fifth, half of total probes with the lowest overall variance [18].

### 2.2. Machine Learning Model

**Figure 1:**
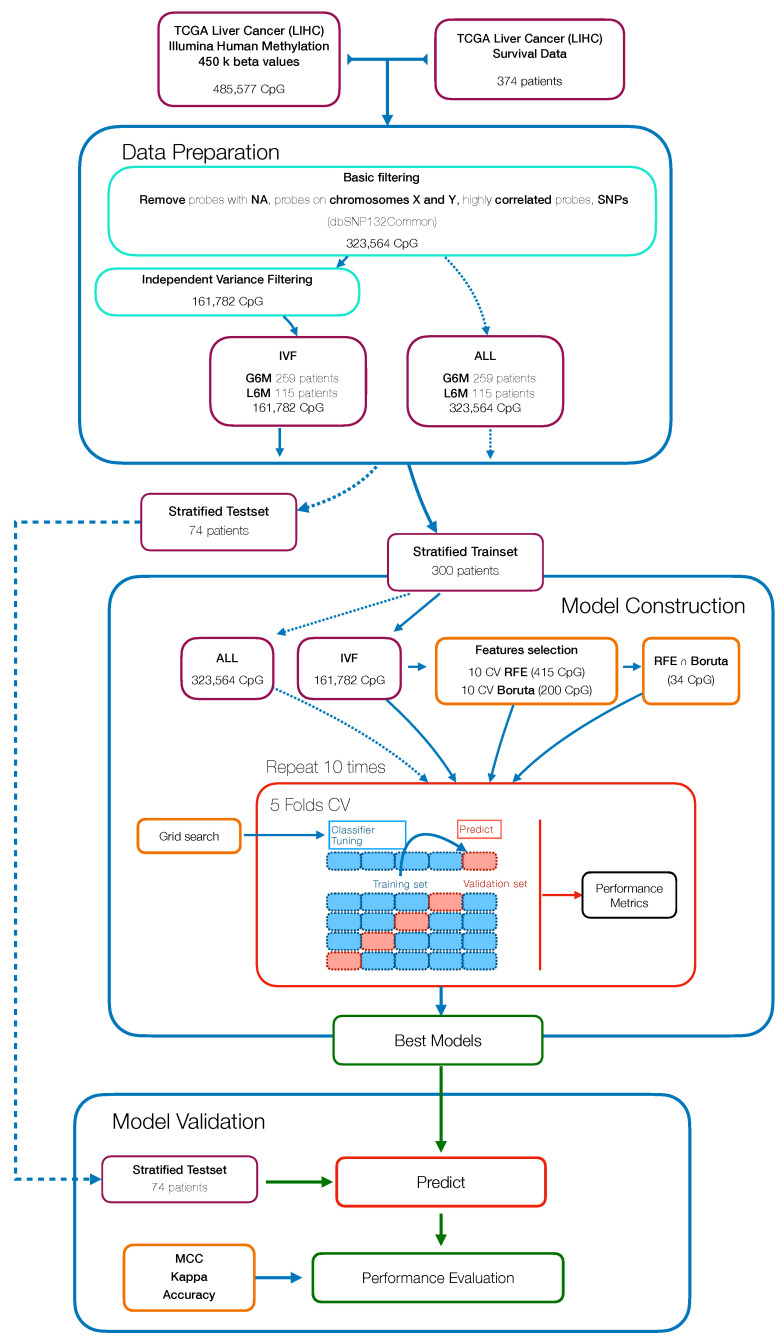
Workflow for the development of a methylation-based Machine Learning model to predict the progression free survival (PFS) status of hepatocellular carcinoma (HCC) patients.

The workflow used to develop and assess the Machine Learning (ML) model for patient classification, with respect to a PFS greater or less than six months, is reported in Figure 1. The major steps include: data preparation and data pre-processing followed downstream by model building, calibration and final validation.

Methylation data were processed and filtered as described in the datasets section. To test whether reducing the number of variables in an unsupervised manner would have improved the classification performance, we started with an independent variance filtered dataset (IVF) (basic filtering + variance filtering) and a basic filtered dataset (ALL). Then both datasets were split into 80% training and 20% test partitions in a stratified manner, making sure that the pair of classes were present in both partitions and in equal proportion. The training set was used in the model construction step while the testset was kept aside to perform the model validation.

Within the model construction step, we evaluated the performance of a supervised learning algorithm namely Random Forest (RF), using the “ranger” package (v0.12.1) [19]. Different analysis pipelines were defined, the algorithm was trained: on all variables (ALL), following a backward features selection (Recursive Features Selection, RFE) (RFE), following an all relevant features analysis (Boruta), and on the overlapping features extracted from these two selection techniques (RFE∩Boruta + RF). The RFE features selection was achieved using the rfe function of “caret” package (v6.0-86) [20], within a 10-fold cross-validation (CV) and using RF algorithm on each iteration to extract the feature importance. The all relevant feature selection was performed with a wrapper algorithm called “Boruta” package (v6.0.0) [21], within a 10-fold CV to assess the best number of features for the output classification.

In order to forcefully assess the pipelines performance, the algorithms were implemented within a 10 × 5 CV scheme, using the train function of caret package; again, the fold assignment was stratified to avoid class imbalances. The hyperparameters optimization was performed within the CV passing a custom grid parameter to the train function.

To avoid overoptimistic inflated results, especially on imbalanced datasets, performance was assessed both in terms of accuracy (ACC) and Matthews Correlation Coefficient (MCC) [22]. The overall performance in cross-validation is evaluated across all CV iterations as average MCC and ACC with 95% studentized bootstrap confidence intervals (CI), and on the test partition as MCC and ACC. To verify the occurrence of possible selection bias effects, the pipeline was also run with randomized output labels.

### 2.3. Correlation Analysis between Methylation Degree and Gene Expression

The CpG sites’ methylation effect on cis regulated gene expression was assessed using Pearson’s correlation (r) test between the CpGs β values and the normalized transcript counts of the corresponding genes [23]. The threshold for a significant correlation was set as |r| > 0.2 and BH adjusted *p*-value < 0.05 [24].

### 2.4. Survival Analysis and Cox Regression Model

Univariate Cox proportional hazards regression analysis was performed using the “survival” package (v3.2-3) [25] to screen which selected CpG probes were associated with patients’ PFS. Afterward, statistically significant (*p*-value adjusted BH < 0.05) CpGs were used to construct a multivariate Cox regression model using a stepwise model selection in both direction, using the stepAIC function from “MASS” package (v7.3-51.6) [26]. The corresponding risk scores of each patient were calculated using the resulted regression model, then samples were stratified into high risk and low risk groups based on the risk cutoff value of 1. Kaplan–Meier (KM) PFS curves were plotted to evaluate the prognostic value of the model using the package “survival”.

### 2.5. Decision Curve Analysis

To assess the adequacy of our CpGs-based prognostic signature and the possible clinical advantage over currently used parameters, we performed a decision curve analysis (DCA) [27] using the R function dca available at http://www.decisioncurveanalysis.org. PFS at 6 months was defined as binary outcome variable (G6M, L6M) and, as predictors, we selected tumor features, biomarkers and the risk score predicted by our CpGs-based prognostic signature.

### 2.6. Differentially Expressed Gene Analysis

To identify DEGs between high risk and low risk groups we used the “DESeq2” package (v1.26.0) [28]. The DEGs analysis was conducted using thresholds of absolute $\log_{2}$fold change (logFC) and adjusted *p*-value (false discovery rate—FDR). Gene expression between the two groups was considered deregulated with a FDR < 0.05 and logFC ≥ 1 or ≤−1, the latter indicating up- and downregulated expression in the high risk group, respectively.

### 2.7. Gene Set Enrichment Analysis

Different enrichment methods were used to analyze the functional characteristics of DEGs between the two groups. Gene Ontology (GO) annotation was performed using “goseq” package (v1.38.0) [29], Disease ontology annotation was performed using “DOSE” package (v3.12.0) [30] and the Kyoto Encyclopedia of Genes and Genomes annotation (KEGG) was performed with “clusterProfiler” package (v3.14.3) [31]. In all methods, an adjusted *p*-value (Benjamini–Hochberg correction, BH) < 0.05 was considered as the threshold level of statistical significance.

### 2.8. Computational and Software Setup

All the analyses were performed in R environment (v3.6.3) [32] on a 12-core Intel Xeon E3-12xx v2 workstation with 72 GB of RAM running Ubuntu 18.04.5 LTS. Graphical plots were created using “ggplot2” package [33] and graphical heatmaps were drawn using “pheatmap” package [34].

## 3. Results

### 3.1. Constructing Prediction Models by Machine Learning

In Figure 1 we present the workflow that resumes the sequence of steps needed to develop and assess the Machine Learning (ML) pipeline to predict the PFS of HCC patients.

The study included 374 primary tumor cases of HCC from The Cancer Genome Atlas (TCGA) cohort. The main clinical and etiological features of cohort used in this study are summarized in Appendix A Table A1. Firstly, patients were stratified in two groups, one comprising patients with a PFS less than six months (L6M) and the other comprising patients with a PFS greater than six months (G6M); the two groups were composed by 115 and 259 patients, respectively.

The CpG beta values from 450k DNA methylation microarray analysis, consisted of 485,577 CpG methylation probes, that were pre-processed by applying different basic filters to remove: probes containing missing values (n = 116,392); highly correlated probes (n = 29,760); probes residing on X and Y chromosomes (n = 12,662); and probes containing single-nucleotide polymorphisms (n = 4798). By using this approach, we obtained a final series of 323,564 probes for the subsequent analyses. We then performed an unsupervised feature selection by an independent variance filtering (IVF) on the half most variable probes (n = 161,782) [18].

**Figure 2:**
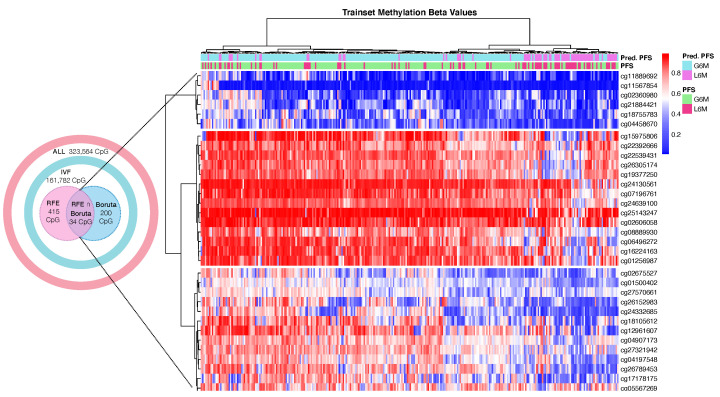
Heatmap of hierarchical clustering of 300 HCC trainset patients by DNA methylation beta values of the final 34 cytosine-phosphate-guanine dinucleotide (CpG) sites. Abbreviations: real progression free survival class (PFS); predicted progression free survival class by the best model (Pred.PFS); greater than six months (G6M); lower than 6 months (L6M). Color scale: *blue* = hypomethylated CpG site; *white* = normal methylated CpG site; *red* = hypermethylated CpG site.

The methylation filtered data were split into training (300 patients) and test (74 patients) sets. The training set was used for the model development and optimization within a 10 × 5 CV and the testset was used for assessing the model performance. In parallel, within the model development step, we used two feature selection methods called RFE and Boruta to reduce the number of features and to possibly identify PFS-specific methylation markers. By using RFE, we got a set of 415 CpG probes that led to the RF algorithm with the highest MCC value. With Boruta analysis, we found that the best subset of probes was the first 200 CpG ranked by importance. We also took in consideration the overlapping probes from the two methods (Figure 2) (Table A3).

The subsets of probes obtained were used to train RF models following the approach illustrated in Figure 1. As reported in Table 1, we tested different pipelines: all basic filtered probes (ALL + RF), independent variance filtered probes (IVF + RF), probes selected using RFE method (RFE + RF), probes selected using Boruta method (Boruta + RF) and overlapping probes from RFE and Boruta (RFE∩Boruta + RF). In Table 1, the results of the classification performance are shown. These results indicated that the dimensionality reduction by feature selection techniques can improve the classification performance in all the experimental groups [35]. In fact, (ALL + RF) and (IVF + RF) achieved a trainset mean cross-validation MCC of 0.127 and 0.162, respectively. In contrast, (RFE + RF), (Boruta + RF) and (RFE∩Boruta + RF) achieved a trainset mean cross-validation MCC of 0.467, 0.485 and 0.533, respectively, that were significantly higher than RF models trained with thousands of features. The trend of increased performance due to dimensional reduction was also seen in the testset partition (Table 1).

Conclusively, the RF model (RFE∩Boruta + RF) showed the best performance metrics and 34 CpG probes (Figure 2) were selected as final predictive markers for PFS prediction.

### 3.2. Correlation of Final CpG Site Methylation and Matched Gene Expression

**Figure 3:**
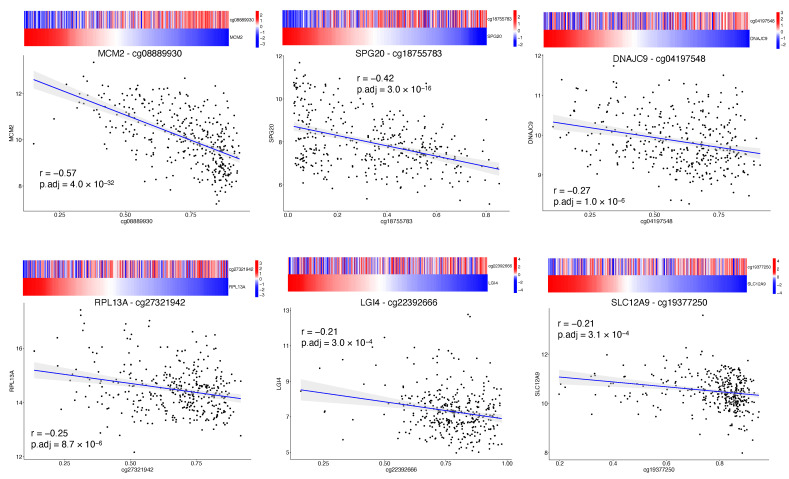
Correlation of CpG site methylation and corresponding gene expression. Matched methylation and gene expression data were plotted to highlight the correlation of the first six (out of 34 final CpG sites) more correlated CpG sites. The x-axes represent the beta value of the probe. The y-axes represents the gene expression reported as variance stabilizing transformation (vst) of raw counts. The heatmap above each plot shows the beta value of the probe and vst counts of the matched gene; color scale depicts standardized transformed values (z-scores).

DNA methylation is an epigenetic mechanism that involves the addition of a methyl group to a DNA cytosine and occurs mainly at CpG dinucleotide sequences in mammals. The degree of methylation at CpG sites but also the balancing of methylated and unmethylated CpGs over the genome control several biological functions such as gene expression regulation, cellular differentiation and mammalian development [36]. Aberrant DNA methylation has been associated with cancer, where the epigenetic reprogramming might play a part in cancer pathogenesis by regulating the expression of tumor suppressor genes or oncogenes [37,38]. We investigated the impact of the 34 final CpG sites on the local regulation of matched genes expression (cis-acting) by conducting Pearson correlation analysis [23]. The analysis was achievable for 26 CpGs due to the lack of matched gene expression for the remaining excluded probes. Among the 26 CpGs, 5 CpG sites showed a weak significant correlation (0.2 <|r| < 0.4, BH adjusted *p*-value < 0.05) and 2 CpG sites showed a moderate significant correlation (0.4 <|r| < 0.7, BH adjusted *p*-value < 0.05) [24] (Figure 3, Table A2). Of note, all significantly correlated CpG sites were characterized by an inverse correlation between DNA methylation and gene expression, that seems to be consistent with the transcriptional silencing effect of DNA methylation [39].

Minichromosome maintenance complex component 2 (*MCM2*) and *SPG20* were the genes that showed a moderate negative correlation with their matched CpG probe. The *SPG20* gene encodes a protein called Spartin that has been found to be involved in intracellular epidermal growth factor receptor trafficking; *SPG20* promoter has been found to be hypermethylated in colorectal cancer, resulting in gene silencing and cytokinesis arrest [40]. *SPG20* promoter hypermethylation was also validated as a novel noninvasive biomarker [41]. *MCM2* is one of six highly conserved proteins recruited to form the MCM protein complex, a cell ubiquitous hexamer that works as molecular motor performing DNA duplex unwinding and fork progression during DNA replication [42]. *MCM2* has been found to be overexpressed in several cancers such as oral, gastric, colon, lung and breast cancer. High *MCM2* expression in cancers was associated with higher grades, more advanced stages and poor prognosis [43].

### 3.3. Construction of CpG-Based Prognostic Signature

To study the possible prognostic impact of the selected CpG probes we used Cox regression analysis to assess which of the 34 final CpG probes were associated with PFS time in the trainset. Eleven CpG probes were significantly correlated with the PFS (*p*-value BH adjusted <0.05) (Table A3). Afterwards, to better identify which of them could be more important in the clinical outcome assessment, a multivariate Cox regression analysis was performed using a stepwise model selection in both direction starting with a model that included all probes. Four CpG probes were identified (Table 2): cg08889930 (*MCM2,TPRA1*), cg11889692 (*TMEM63C,RP11-463C8.4*), cg12961607(*SRSF7*), cg22539431 (*SND1*). Three CpG sites (cg08889930,cg12961607,cg22539431) were characterized by a negative coefficient that in this case implies a higher PFS for patients with higher beta values of these CpG sites; conversely one site (cg11889692) with a positive coefficient confers a lower PFS for patients with higher beta values of this CpG site. Importantly, lower values of cg08889930 were associated with a worse PFS and this is in keeping with the evidence that we reported that its value was negatively correlated with *MCM2* expression. The hypomethylation of cg08889930 and the resultant overexpression of *MCM2* were in keeping with what was previous reported [43].

Taking into account the estimated Cox regression coefficients, we then constructed a prognostic risk model described by the formula as follows:

$$\begin{array}{cc} RiskScore= & \left(-1.796)\times cg08889930(\beta value)+(1.448)\times cg11889692(\beta value\right)\\ \; & +(-0.852)\times cg12961607(\beta value)+(-1.870)\times cg22539431(\beta value) \end{array}$$

**Figure 4:**
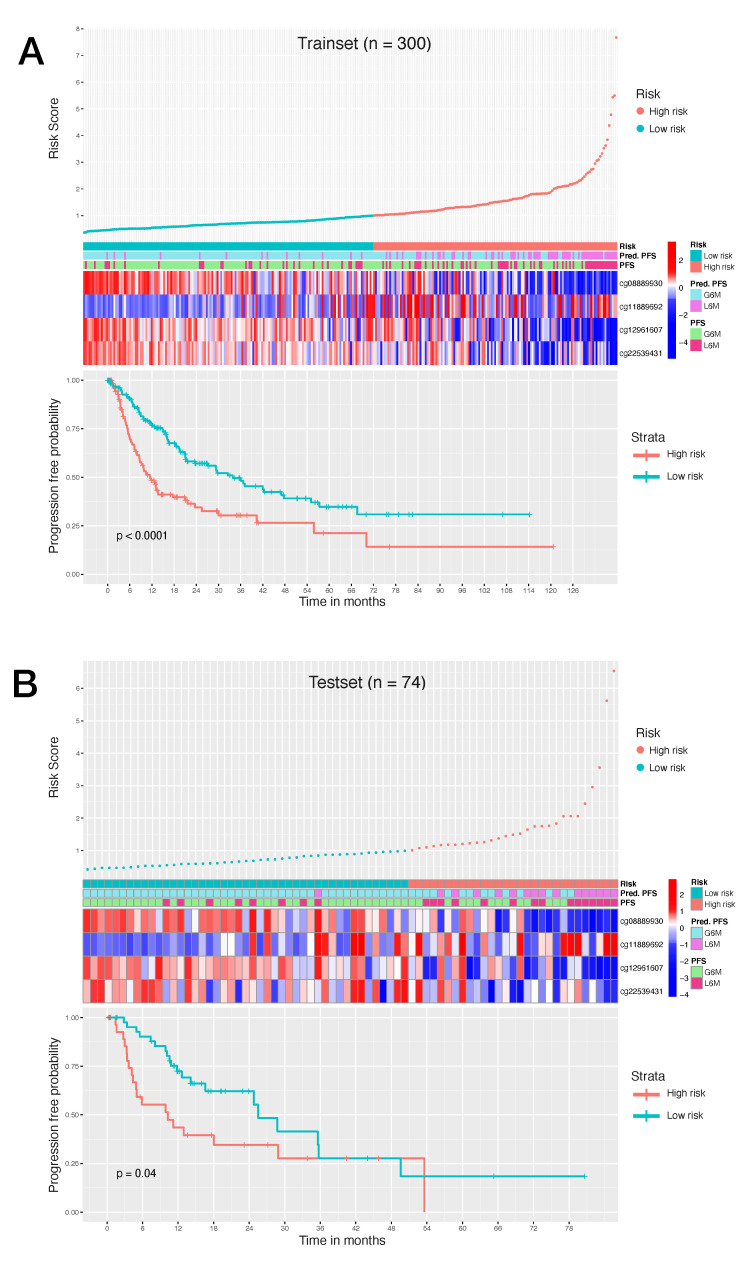
(**A**): Survival analysis of 300 HCC trainset patients. (**B**): Survival analysis of 74 HCC testset patients. Top graph shows the patient risk score calculated by the signature, by stratifying patients in high risk of progression and low risk of progression. Middle heatmap shows the calculated risk contribution of each CpG; patients are ordered in accordance with risk score (color scale is Z-score of absolute risk values). Bottom plot shows Kaplan–Meier PFS curves for high risk and low risk patients.

The corresponding risk scores of each trainset patient were calculated using the formula and the samples were stratified into high-score and low-score groups based on the risk score = 1 as cutoff. Next, we used Kaplan–Meier survival analysis to assess the prognostic impact of the model in the risk stratified trainset. The relationship between PFS time and risk score was statistically significant, with patients at high risk of progression (n = 137) showing a considerable adverse PFS with respect to the patients at low risk of progression (n = 163). This trend appeared as particularly visible during the first 12 months (Figure 4A). Patients at high risk of progression had a significantly increased risk of disease progression.

To validate the risk model, we also tested the established model in the testset. The risk scores were calculated using the aforementioned formula and the patients were stratified in patients at high risk of progression (n = 29) and patients at low risk of progression (n = 45). Low risk patients showed a significant advantage in PFS time compared to high risk patients (Figure 4B). These results demonstrated that our model can provide an accurate risk stratification system and reveal that the methylation level of these CpG probes could affect the prognosis of HCC patients.

### 3.4. Alpha-Fetoprotein Level in Risk Stratified Patients

**Figure A1:**
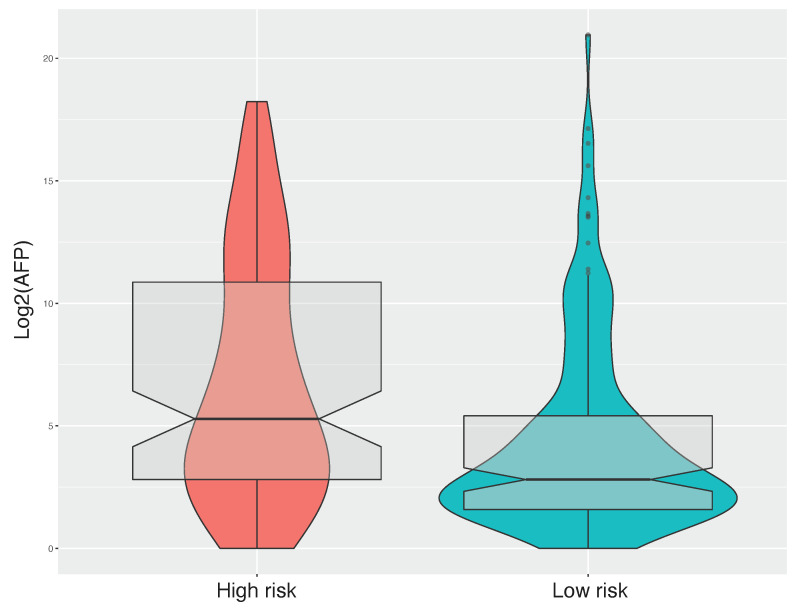
Alpha-fetoprotein boxplot of risk groups. Preoperative alpha-fetoprotein levels are reported on the Y-axis as base-2 log scale of preoperative alpha-fetoprotein value.

Alpha-fetoprotein (AFP) is the first introduced and most extensively utilized marker for diagnosis, prognosis and monitoring of HCC [44]. To test if preoperative AFP concentration is higher in patients at high risk of progression, we analyzed its distribution between high risk and low risk groups (Figure A1). Preoperative alpha-fetoprotein concentration in high risk patients was statistically higher than low risk patients (Unpaired two-samples Wilcoxon test, *p*-value = $8.347\times 10^{-6}$). This result was consistent with the fact that AFP level has been demonstrated to be an independent risk predictor associated with pathological grade, progression and survival of HCC [45].

### 3.5. Decision Curve Analysis

Decision curve analysis (DCA) is a method that can be used to assess the value of prognostic models [27]. In brief, DCA calculates a clinical “net benefit” for one or more predictors, or diagnostic models, in comparison to reference strategies that are treating all patients or treating no patients.

A number of models have been developed to calculate the tumor stage and prognosis. Since the important role of the liver, HCC evaluation includes not only tumor features but also liver function parameters as key prognostic factors for survival and progression. Different staging algorithms are currently in use [46], they usually include prognostic clinical variables, tumor burden variables, liver function variables and biomarkers.

**Figure 5:**
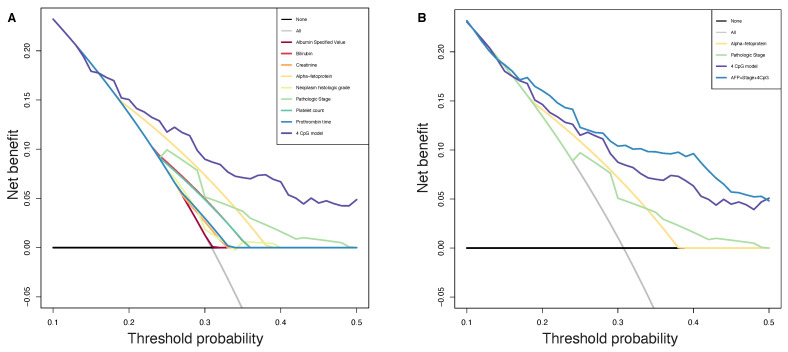
Decision curve analysis. The plot shows the net benefit per patient relative to no intervention for any patient (“treat none”). The unit is the benefit for a patient who would have a PFS less than 6 months without intervention and who receives the intervention. Black line: assume no patient has a PFS less than 6 months. Grey line: assume all patients have a PFS less than 6 months. (**A**): All parameters are reported, purple line representing our CpGs based method achieving higher benefit than in-use parameters. (**B**): Pathologic stage, preoperative AFP, 4 CpGs risk score and model including all these three variables are reported, blue line shows that the benefit was even higher when we combined our model to strong prognosis factors.

We analyzed the predicted probability of PFS at six months using the risk calculated by our 4 CpGs model and by common in-use clinical parameters, to assess the net benefit that patients could obtain. As shown in Figure 5A, our method showed more benefits with respect to in-use parameters, implying that our 4 CpG model performs well and is able to stratify patients with PFS greater or lower than six months. Moreover, the benefit was even more higher when we combined our model to strong prognosis factors such as preoperative AFP levels and AJCC pathologic stage (Figure 5B).

### 3.6. Identification of DEG Genes between Risk Stratified Patients

**Figure 6:**
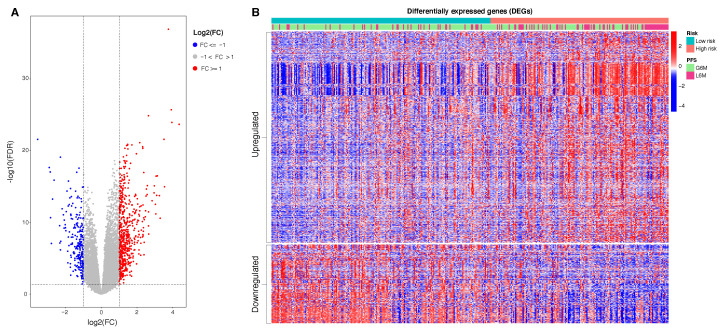
(**A**): Volcano plot of DEGs: in *red* 619 upregulated genes, in *blue* 231 downregualted genes. (**B**): Heatmap of 850 DEGs. The data represent standardized fold change values. Patients in columns are ordered in accordance with calculated risk score. The genes in rows are clustered in upregulated and downregulated genes found in high risk patients compared to low risk patients. Abbreviations: real progression free survival class (PFS); greater than six months (G6M); lower than 6 months (L6M). Color scale: from *blue* to *red* = z-scores of normalized transcript count values.

With the aim to understand the functional significance of the proposed epigenetic 4 CpGs score, we evaluated the putative gene expression modulation between the two epigenetic risk categories. To do this, we performed a DEG analysis. Patients at low risk of progression were used as reference. A total of 850 DEGs were identified, consisting of 619 upregulated genes (FDR < 0.05 and logFC ≥ 1) and 231 downregulated genes (FDR < 0.05 and logFC ≤ 1). In Figure 6, we reported the heatmap of DEGs in which patients are ordered in accordance with the calculated risk score. Of note, as the risk score increases (from the left to the right of the heatmap), the fold change increases in upregulated genes and decreases in downregulated genes. In Table A4, we reported the top 25 upregulated and downregulated genes in patients at high risk of progression compared to patients at low risk of progression.

### 3.7. Functional Enrichment Analysis of DEGS

**Figure 7:**
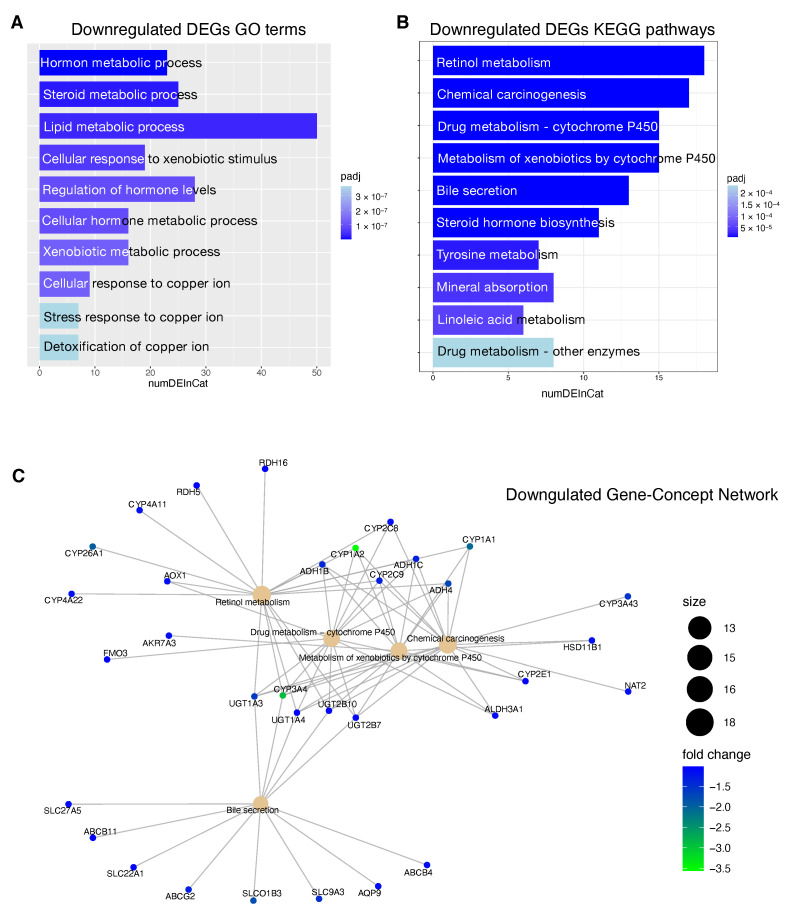
Functional enrichment analysis of downregulated DEGSs in patients at high risk of progression. (**A**): Top 10 GO terms plotted in order of adjusted *p*-values (BH). Bar size represents the number of significant DEGs that fall within a GO category *(numDEInCat)* and color represents the adjusted *p*-values (BH). (**B**): KEGG pathways are ordered by adjusted *p*-values (BH), bars size represent the number of significant DEGs that fall within a KEGG pathway *(numDEInCat)* and color represents the adjusted *p*-values (BH). (**C**): Gene-Concept Network. The size of the KEGG pathways stands for the number of DEGs that fall within each pathway. Color scale of gene names stands for the $\log_{2}$-fold change of DEGs in the high risk of progression group compared to the low risk of progression group.

**Figure 8:**
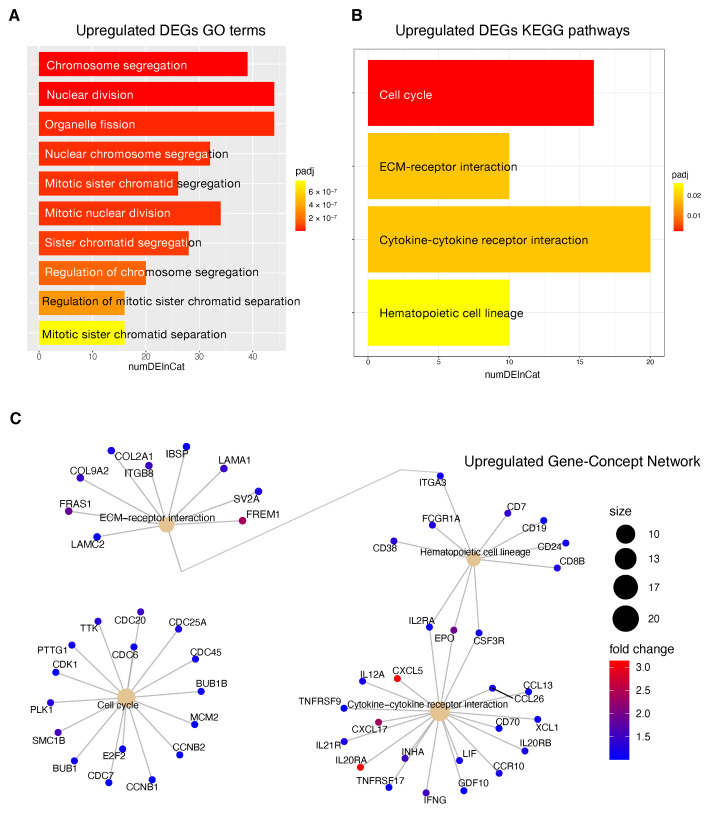
Functional enrichment analysis of upregulated DEGs in patients at high risk of progression. (**A**): Top 10 GO terms plotted in order of adjusted *p*-values (BH). Bar size represents the number of significant DEGs that fall within a GO category *(numDEInCat)* and color represents the adjusted *p*-values (BH). (**B**): KEGG pathways are ordered by adjusted *p*-values (BH), bar size represents the number of significant DEGs that fall within a KEGG pathway *(numDEInCat)* and color represents the adjusted *p*-values (BH). (**C**): Gene-Concept Network. The size of the KEGG pathways stands for the number of DEGs that fall within each pathway. Color scale of gene names stands for the $\log_{2}$-fold change of DEGs in the high risk of progression group compared to the low risk of progression group.

In order to gain more insights into the biological function of the risk prediction signature, we applied GO terms and KEGG pathway analyses to identify associated biological terms and pathways from DEG profiles of risk stratified HCC patients. The results of the GO analysis revealed that downregulated DEGs in patients at high risk of progression were significantly enriched in biological processes including ‘hormone metabolic process’, ‘lipid metabolic process’, ‘xenobiotic metabolic process’ and ‘cellular response to xenobiotic stimulus’ (Figure 7A). The upregulated DEGs in patients at high risk of progression were mainly enriched in ‘chromosome segregation’, ‘nuclear division’ and ‘mitotic nuclear division’ Figure 8A).

KEGG pathway analysis revealed that downregulated DEGs in high risk patients were associated with pathways including ‘Retinol metabolism’, ‘Chemical carcinogenesis’ and ‘Drug metabolism—cytochrome P450 ’ (Figure 7B). The upregulated DEGs high risk patients were enriched in ‘Cell cycle’, ‘ECM-receptor interaction’, ‘cytokine receptor interaction’ and ‘Hematopoietic cell lineage’ Figure 8B).

In Figure 7C and Figure 8C, we reported the Gene-Concept Network of the main downregulated and upregulated KEGG pathways in patients at high risk of progression. Downregulated genes included several clusters of metabolic genes such as: alcohol dehydrogenases (*ADH*) genes that are involved in alcohol metabolism, uridine diphosphate glucuronyl transferase (*UGT*) genes which play roles in phase II drug metabolism and cytochromes (*CYPs*) P450 genes that catalyze the oxidation and metabolism of a large number of xenobiotics and endogenous compounds. Within downregulated genes, we also found solute carriers (*SLC*) and ATP-binding cassette (*ABC*) proteins that play an essential role in the uptake and in the export of a large variety of anti-tumor drugs, respectively.

Upregulated genes included several genes involved in the control of the cell cycle like *E2F2*, cyclin-dependent kinase 1 (*CDK1*) and *MCM2* but also cytokine gene network with crucial effects on inflammation and tumor immunology as well. Within upregulated genes we found surface markers that are expressed by stage- and lineage-specific hematopoietic cell; as an example, overexpression of *CD19*, *CD24* and *CD38* could indicate an higher infiltration level of B regulatory cells (Bregs) with a $CD19^{+}$$CD24^{hi}$$CD38^{hi}$ phenotype. Breg phenotype has been found to be enriched in the tumor microenvironment and to be associated with progression of several cancers, including HCC [47,48,49].

## 4. Discussion

HCC remains one of the most frequent malignancies and a leading challenge for public health worldwide. Even after the application of curative treatments, such as resection, local ablation and liver transplantation, and the extensive advancement in earlier diagnosis, staging systems and therapy decision-making, the long-term prognosis of HCC remains poor. The evaluation of conventional parameters such as proper liver function, vessel invasion, tumor staging and biomarker levels are commonly used in HCC prognosis prediction; nevertheless, their performance is still not satisfactory and this could be due to the high degree of heterogeneity among HCC cases. Therefore, the recognition of novel prognostic biomarkers from large scale omics data and the establishment of more accurate prognostic models could dynamically recapitulate the biological progression of HCC and may have a superior predictive accuracy than conventional ongoing parameters. Recently, numerous studies with gene expression, microRNAs and methylation profiling have shown great potential in prognosis prediction and staging systems of HCC [50,51,52,53,54]. The main goal of this strategy is to stratify patients into homogeneous prognosis clusters, which then can provide the bases for the option of most appropriate interventions. Although several studies have been conducted, many perspectives still remain unexplored. A prediction model that can reliably classify HCC patients into homogeneous groups with respect to the PFS time can be of particular importance in the context of HCC where the disease progression is still not entirely definable by conventional prognosis parameters in the attempt to recapitulate prognosis and treatment response.

The present study aimed to identify efficient prognostic markers to stratify HCC patients according to their epigenetic features involved in tumor pathogenesis and progression. We applied a combination of Machine Learning algorithms that performed a supervised features selection (i.e., Boruta, RFE) on methylation data to increase the chances of catching CpG markers related to the PFS [55,56]. The subsets of probes obtained were used to train RF models to select the best predictive model resulting in a final 34 CpGs-based model.

One of the advantages of RF is the built-in variable importance measure that ranks the features with respect to their relevance for prediction. This is performed calculating the Gini Importance (Mean Decrease in Impurity—MDI) [57] or the Permutation Importance (Mean Decrease in Accuracy—MDA) that can correct the Gini importance bias [58]. However, techniques that estimate the variable importance are not able to capture patterns of dependency between features and response; they only represent the strength of this dependency as a single number, and the obtained results can be difficult to interpret [59].

Consequently, to evaluate the size and direction of the relationship between the 34 CpG features and the PFS, but also to investigate their prognostic impact, we firstly performed the univariate Cox regression analysis of the final 34 markers and then, we established a 4-CpG-based prognostic model for HCC. The signature was validated using the internal left-out testing set, indicating the stability of this model in terms of ability to stratify HCC patients into high and low risk groups for early cancer progression. To assess the adequacy and the clinical advantages of our signature over currently used parameters, we performed a decision curve analysis proving that our method showed a significantly improved performance when compared to in-use conventional clinical parameters (i.e., preoperative AFP levels and AJCC pathologic stage), thus indicating a more powerful and dynamic reflection of HCC heterogeneity. This was true either if the 4 CpG signature was considered alone or in combination with well-recognized prognosis parameters. The dataset considered in this analysis is the most comprehensive collection of multi-omic data from HCC cases [53]. Unfortunately, we were not able to find larger datasets to integrate our analysis. This could be considered as a limitation even if in the attempt to address the lack of an independent validation set, we followed the recommendations described in Shi et al. [60]. The approach presented here relies on the analysis of methylation data. This design has several advantages: the DNA methylation level can be easily accessible in circulating tumor DNA (ctDNA) allowing a noninvasive ‘liquid biopsy’; unlike methods based on somatic alterations analysis in ctDNA, methylation methods do not need the identification of somatic mutations in the tumor; the collection of peripheral blood to obtain cfDNA is less invasive compared with tumor biopsy. However, further studies are needed to validate the feasibility of using the defined CpG sites from ctDNA to guide a personalized approach for HCC patients.

Our CpGs signature comprises the methylation level of 4 CpG sites (Table 2), of which the cg08889930 was also correlated with *MCM2* expression (Figure 3); this site likely represents the methylation status of *MCM2* gene and so an indicator of *MCM2* expression. High risk for progression is driven by a lower level of cg08889930 methylation that results in an overexpression of *MCM2*, which in turn is associated with cancer progression and poor prognosis [43]. Aberrant DNA methylated-differentially expressed genes and pathways in HCC have been previously reported [61,62]. Our epigenetic Machine Learning model better discriminated among previously reported methylated genes, pointing out MCM2 methylation as a key point to define risk of progression for HCC patients. *MCM2* belongs to the minichromosome maintenance (MCM) protein complex which is involved in the initiation of DNA replication and DNA unwinding [42]. *MCM2* is a subunit/component of the hexameric protein complex that consists of *MCM2-7* and directly interacts with *MCM5* [63]. In vitro studies indicate that *MCM2* silencing inhibits cell proliferation by affecting the G1/S transition and conversely the overexpression of *MCM2* promotes cell proliferation in lung cancer cells [64]; moreover, *MCM2* knockdown inhibits cell migration in lung cancer cells. Notably, during the last few years, several studies have evaluated the action of in use treatments against *MCM2*. In particular, *MCM2* is a therapeutic target of Trichostatin A in colon cancer cells [65], *MCM2* has been proposed as therapeutic target of lovastatin in human non-small cell lung carcinomas [66], the combination of *MCM2* silencing and carboplatin treatment may represent a novel therapeutic strategy to treat ovarian cancer [67]. Here we propose that the CpG site cg08889930 could represent the methylation status of the *MCM2* gene and consequently the expression level of *MCM2*. Its mehylation level could serve not only as prognostic marker to evaluate HCC patient progression but also as predictive marker to evaluate the efficacy of a therapeutic intervention. Another important implication is that the MCM complex offers a fascinating target for drug development in HCC, since it is an essential replication factor that couples DNA replication to both cell cycle progression and checkpoint regulation [68].

Information relative to the potential implication for the development of novel therapies [11,69], was also highlighted by the transcriptome analysis for the HCC patients stratified according to the proposed 4-CpG signature. In particular, from the functional enrichment analysis, we found that upregulated genes in the context of the high risk HCC patient group are implicated in important pathways Figure 8C) such as extracellular matrix (ECM) receptor interaction, cell cycle, cytokine receptor interaction and hematopoietic cell lineage. Of note, we also found that *MCM2* is upregulated in patients belonging to the high risk group and this could be at least in part recapitulated by the enrichment of genes involved in the control of DNA replication, cell division and cell cycle (Figure 8). One of the most common features of cancer is cell cycle deregulation which leads to unscheduled proliferation and genomic instability. In humans, the cell cycle is controlled by a subfamily of cyclin-dependent kinases (*CDKs*) and several modulators [70]. Results indicate that *CDK1* is the only CDK that is essential for cell cycle progression [71]. Here, the high risk HCC patient group has been found to be characterized by the upregulation of several effectors that play a role within the *CDK1* network and that are usually upregulated in cancer (Figure 8). Overexpression of *CDK1*, *MCM2*, *E2F2*, *PLK1*, *CCNB1/2*, *BUB1*, *BUB1B*, *CDC25* has been associated with aberrant proliferation in many cancer types [72] including HCC [73]. This could be of interest for the designing of inhibitors of cell cycle protein pathways to be used as anticancer drugs.

One of the major constituents of the environment is the extracellular matrix (ECM). The ECM supervises crucial processes like intratumoral signaling, transport mechanisms, metabolisms and immunogenicity. For its activity, the ECM has been associated with tumor establishment, disease progression and therapy resistance in several tumors [74]. The ECM aberration plays also a role in the carcinogenesis and progression of HCC [75]. In this context, the overexpression of ECM-receptor interactor accelerates liver cancer cell metastasis in vessel and settlement in metastatic sites [76]. In the present study, we found a high expression of some upregulated ECM interactors (Figure 8C) that have been correlated to migration and invasion (*LAMC2*, *FRAS1*) [77,78], drug resistance (*ITGB8*) [79] and proliferation (*LAMA1*) [80].

Tumor progression is promoted by the crosstalk of different cells populations within the tumor microenvironment (TME) and this communication is guaranteed by the release of key mediators such as cytokines and chemokines. These signaling molecules and their receptors affect multiple processes including tumor cell proliferation, invasion and metastasis, tumor immune response and angiogenesis [81]. In this context, in the high risk HCC patient group, the most upregulated genes within the cytokines-chemokines network were *CXCL5*, *CXCL17* and *IL20RA* (Figure 8C). *CXCL5* overexpression promotes HCC cell proliferation, invasion and intratumoral neutrophil infiltration [82]. Moreover, a high *CXCL17* expression and a higher rate of tumor-infiltrating CXCL17-expressing cells have been found to be associated with unfavorable prognosis in HCC patients [83].

The downregulated genes in high risk HCC patients include several metabolic genes, drug metabolism genes, transporters and carriers genes (Figure 7). This scenario has important implications for the choice of an appropriate chemotherapy or an appropriate adjuvant chemotherapy. Until 2007, no effective therapies were available for HCC patients that failed to be treated with locoregional approaches. Between 2007 and 2016, sorafenib was the only systemic drug approved for advanced HCC. Currently, palliative treatment strategies in patients with advanced HCC comprise new pharmacological therapies based on inhibitors of tyrosine kinases (TKIs) like sorafenib and regorafenib, but also classical chemotherapeutic agents and novel immunotherapy strategies [84]. However, several mechanisms of chemoresistance (MOC) [9] can significantly affect the response of HCC patients to the currently used pharmacological treatments. We found that patients at high risk of progression are characterized by the deregulation of some genes involved in mechanisms of chemoresistance (*SLC22A1*, *SLCO1B3*, *ABCG2*, *CYP3A4*). *SLC22A1* is a member of the solute carriers (SLC) family, a collection of proteins that play an essential role in the uptake of anticancer drugs. Loss of *SLC22A1* in the plasma membrane of tumor cells results in a reduction of sorafenib uptake, that has been correlated with less favorable prognosis of HCC patients treated with this drug [85]. Furthermore, restoring *SLC22A1* expression results in an improved receptivity of sorafenib in HCC cells [86]. Another dowregulated gene is *SLCO1B3*, a component of a family of genes that plays a role in the transport of TKIs drugs. *SLCO1B3* accomplishes the uptake of cabozantinib [87], clears sorafenib glucuronidated metabolites [88] and its downregulation in HCC patients contributes to chemoresistance [89]. The ATP-binding cassette *ABCG2* plays a crucial role in sorafenib efflux and higher *ABCG2* expression has also been correlated with chemoresistance in HCC and reduced overall survival in HCC patients [90]. In our study, high risk HCC patients are characterized by the downregulation of the gene *ABCG2*, so in this case the higher expression level could mainly affect the sorafenib behavior in low risk HCC patients. Several CYPs genes are deregulated in high risk patients including crucial genes such as *CYP3A4*, *CYP2C9*, *CYP1A2* (Figure 7C). Deregulated expression of these genes involved in drug and xenobiotics metabolism may affect prodrug activation or drug inactivation, both leading to a lower bioavailability of the functional drug. Sorafenib, regorafenib, cabozantinib and lenvatinib are metabolized by *CYP3A4* through an oxidation process [91,92,93]. Downregulation of *CYP3A4* in HCC microsomes leads to a significantly altered sorafenib metabolism in the liver tumor tissue of HCC patients [94].

## 5. Conclusions

In conclusion, starting from the methylation profile of primary HCC specimens, we used a novel combination of Machine Learning algorithms to capture early tumor progression features and to focus on relevant CpG sites. These final features laid the foundations for the development of a prognostic model for early HCC progression based on 4 CpG sites that showed a significantly improved performance over conventional clinical parameters. Notably, we proposed the oncogenic *MCM2* gene as a methylation-driven gene of which the representative CpG site cg08889930 could serve as a predictive marker of therapeutic interventions. Finally, we provided evidence that our model is capable to classify HCC patients into high and low risk for progression groups. Thus, this predictive tool may enhance the management of patients at high risk of progression and the development of personalized treatment for HCC patients.

## Figures and Tables

**Table 1:** Models performances in cross-validation (mean with confidence intervals) and on the testset. ACC: accuracy; MCC: Matthews Correlation Coefficient; CI: 95% studentized bootstrap confidence interval.

Workflow	Features Selection	N° Features	Hyperparameters	Train Metrics			Test Metrics		
				MCC (CI)	Kappa (CI)	ACC (CI)	MCC	Kappa	ACC
**ALL + RF**	None	323,564	max.depth = 10 num.trees = 50 mtry = 569 min.node.size = 20	0.127 (0.09–0.163)	0.113 (0.081–0.145)	0.679 (0.668–0.690)	0.157	0.120	0.695
**IVF + RF**	IVF	161,782	max.depth = 15 num.trees = 100 mtry = 402 min.node.size = 20	0.162 (0.128–0.197)	0.146 (0.115–0.178)	0.679 (0.665–0.694)	0.138	0.115	0.686
**RFE + RF**	IVF + RFE	415	max.depth = 10 num.trees = 500 mtry = 24 min.node.size = 20	0.467 (0.431–0.503)	0.455 (0.419–0.491)	0.784 (0.771–0.798)	0.428	0.371	0.773
**Boruta + RF**	IVF + Boruta	200	max.depth = 15 num.trees = 200 mtry = 17 min.node.size = 20	0.485 (0.453–0.518)	0.473 (0.440–0.506)	0.790 (0.777–0.803)	0.415	0.394	0.767
**RFE∩Boruta + RF**	IVF + Intersect (RFE-Boruta)	34	max.depth = 15 num.trees = 500 mtry = 5 min.node.size = 20	0.533 (0.502–0.563)	0.523 (0.493–0.553)	0.806 (0.794–0.818)	0.510	0.484	0.802
**RFE∩Boruta + RF (randomized output)**	IVF + Intersect (RFE Boruta)	34	max.depth = 15 num.trees = 500 mtry = 5 min.node.size = 20	0.018 (−0.016–0.053)	0.014 (−0.010–0.037)	0.671 (0.663–0.680)	−0.065	−0.042	0.648

**Table 2:** Coefficients of the four CpGs multi-variate cox regression model. Abbreviations: HR = hazard ratio; CI = confidence interval; * = *p*-Value < 0.05; ** = *p*-Value < 0.01.

CpG	Gene	Coeff. b${}_{i}$	HR [exp(b${}_{i}$)]	HR 95%CI	*p*-Value	Significance
**cg08889930**	*MCM2, TPRA1*	−1.796	0.1660	(0.05–0.52)	0.00222	**
**cg11889692**	*TMEM63C, RP11-463C8.4*	1.448	4.2541	(1.79–10.10)	0.00104	**
**cg12961607**	*SRSF7*	−0.852	0.4265	(0.19–0.94)	0.03573	*
**cg22539431**	*SND1*	−1.870	0.1541	(0.04–0.59)	0.00626	**

## Data Availability

All the data showed are based on data from TCGA Research Network: https://www.cancer.gov/tcga. Further information are available upon request.

## Abbreviations

The following abbreviations are used in this manuscript:

ACC	Accuracy
AFP	Alpha-fetoprotein
AJCC	American Joint Committee on Cancer
BH	Benjamini–Hochberg
CpG	Cytosine-phosphate-guanine dinucleotide
CV	Cross-validation
DCA	Decision curve analysis
DEGs	Differentially expressed genes
ECM	Extracellular matrix
FC	Fold change
FDR	False discovery rate
G6M	Greater than six months
GDC	Genomic Data Commons
GO	Gene Ontology
HCC	Hepatocellular carcinoma
IVF	Independent variance filtered
KEGG	Kyoto Encyclopedia of Genes and Genomes annotation
KM	Kaplan–Meier
L6M	Less than six months
LIHC	Liver Hepatocellular Carcinoma
MCC	Matthews Correlation Coefficient
ML	Machine Learning
MOC	Mechanisms of chemoresistance
PFS	Progression-free survival
RF	Random Forest
RFE	Recursive Features Selection
TCGA	The Cancer Genome Atlas
TME	Tumor microenvironment
TNM	Tumor, Node, Metastasis

## Appendix A

**Table A1:** Clinical and etiological features of the HCC cases entering the study cohort.

Variable		Allset (n = 374)
Age (mean ± SD)		59.4 ± 13.5
PFS (%)		
	G6M	259 (69.3)
	L6M	115 (30.7)
Gender (%)		
	Male	253 (67.6)
	Female	121 (32.4)
Race (%)		
	White	187 (50)
	Asian	160 (42.8)
	Black	17 (4.5)
	Not reported	10 (2.7)
Alcohol consumption (%)		
	Yes	118 (31.6)
	No	256 (68.4)
Hepatitis (%)		
	Hepatitis B	107 (28.6)
	Hepatitis C	56 (15)
Other (%)		
	Hemochromatosis	6 (1.6)
	Non-Alcoholic Fatty Liver Disease	20 (5.3)
	No History of Primary Risk Factors	111 (29.7)
	Other	12 (3.2)

**Table A2:** Correlation of CpG methylation and matched gene expression. Abbreviations: *r* = Pearson correlation coefficient; Adj. *p*-value = Benjamini–Hochberg adjusted *p*-value.

CpG	Gene	r	Adj. *p*-Value	Interpretation	Direction
cg08889930	*MCM2*	−0.57	$4.00\times 10^{-32}$	moderate	negative
cg18755783	*SPG20*	−0.42	$3.00\times 10^{-16}$	moderate	negative
cg04197548	*DNAJC9*	−0.27	$1.00\times 10^{-6}$	weak	negative
cg27321942	*RPL13A*	−0.25	$8.70\times 10^{-6}$	weak	negative
cg19377250	*SLC12A9*	−0.21	$3.10\times 10^{-4}$	weak	negative
cg21884421	*IGDCC3*	−0.21	$3.00\times 10^{-4}$	weak	negative
cg22392666	*LGI4*	−0.21	$3.00\times 10^{-4}$	weak	negative
cg07196761	*PAAF1*	−0.19	$8.40\times 10^{-4}$	negligible	negative
cg26152983	*MAGEF1*	−0.19	$8.40\times 10^{-4}$	negligible	negative
cg05567269	*TJP1*	−0.17	$2.40\times 10^{-3}$	negligible	negative
cg02360980	*LTK*	−0.16	$6.00\times 10^{-3}$	negligible	negative
cg04907173	*POLA2*	−0.16	$6.00\times 10^{-3}$	negligible	negative
cg22392666	*FXYD7*	−0.15	$7.30\times 10^{-3}$	negligible	negative
cg01500402	*MLST8*	−0.14	$1.60\times 10^{-2}$	negligible	negative
cg26789453	*ERP29*	−0.13	$2.40\times 10^{-2}$	negligible	negative
cg24639100	*DNAJC14*	−0.11	$6.70\times 10^{-2}$	negligible	negative
cg11567854	*NR4A1*	−0.09	$1.30\times 10^{-1}$	negligible	negative
cg26789453	*TMEM116*	−0.09	$1.30\times 10^{-1}$	negligible	negative
cg11889692	*TMEM63C*	−0.08	$1.70\times 10^{-1}$	negligible	negative
cg26305174	*TRIP6*	−0.08	$2.00\times 10^{-1}$	negligible	negative
cg02606058	*FBXL8*	−0.07	$2.90\times 10^{-1}$	negligible	negative
cg17178175	*NFE2L2*	−0.06	$3.80\times 10^{-1}$	negligible	negative
cg22539431	*SND1*	−0.03	$6.50\times 10^{-1}$	negligible	negative
cg08889930	*TPRA1*	0	$9.70\times 10^{-1}$	zero	zero
cg26305174	*SLC12A9*	0	$9.70\times 10^{-1}$	zero	zero
cg16224163	*LPP*	0.02	$6.90\times 10^{-1}$	negligible	positive
cg02675527	*PAX8*	0.03	$6.40\times 10^{-1}$	negligible	positive
cg24130561	*DICER1*	0.03	$6.40\times 10^{-1}$	negligible	positive
cg01256987	*GXYLT1*	0.04	$5.20\times 10^{-1}$	negligible	positive
cg02606058	*TRADD*	0.04	$5.50\times 10^{-1}$	negligible	positive
cg25143247	*PACRG*	0.17	$2.40\times 10^{-3}$	negligible	positive

**Table A3:** Univariate Cox analysis of the 34 final selected CpG. Abbreviations: HR = hazard ratio; CI = confidence interval; Adj. *p*-value = Benjamini–Hochberg adjusted *p*-value.

CpG	Gene	Coeff. b${}_{i}$	HR [exp(b${}_{i}$)]	HR 95%CI	*p*-Value	Adj. *p*-Value
cg16224163	*LPP-AS2, LPP*	−1.970	0.14	(0.06–0.35)	<0.0001	0.0007
cg24130561	*DICER1*	−2.773	0.06	(0.02–0.24)	<0.0001	0.0007
cg08889930	*MCM2, TPRA1*	−2.031	0.13	(0.05–0.38)	0.0002	0.0015
cg12961607	*SRSF7*	−1.428	0.24	(0.11–0.51)	0.0002	0.0015
cg07196761	*COA4, PAAF1*	−2.640	0.07	(0.02–0.29)	0.0002	0.0016
cg22539431	*SND1*	−1.907	0.15	(0.04–0.49)	0.0018	0.0103
cg26789453	*ERP29*, *TMEM116*	−1.176	0.31	(0.14–0.69)	0.004	0.0193
cg01256987	*GXYLT1*	−1.485	0.23	(0.08–0.67)	0.0073	0.0278
cg06496272	*AC005682.5*, *SNORD93*	−1.204	0.30	(0.13–0.72)	0.007	0.0278
cg11889692	*TMEM63C*, *RP11-463C8.4*	1.075	2.93	(1.27–6.74)	0.0115	0.0389
cg26152983	*MAGEF1*	−0.930	0.39	(0.19–0.82)	0.0126	0.0389
cg15975806	.	−0.915	0.40	(0.18–0.88)	0.0224	0.0586
cg17178175	*NFE2L2*	−0.843	0.43	(0.21–0.88)	0.0213	0.0586
cg02606058	*FBXL8*, *TRADD*	−1.719	0.18	(0.04–0.82)	0.0269	0.0639
cg24639100	*DNAJC14*, *RP11-762I7.5*	−1.514	0.22	(0.06–0.85)	0.0282	0.0639
cg04197548	*DNAJC9*	−1.058	0.35	(0.13–0.91)	0.0319	0.0679
cg01500402	*MLST8*	−1.471	0.23	(0.05–0.99)	0.048	0.0959
cg04458670	*ICE1*	−0.771	0.46	(0.2–1.06)	0.0676	0.1276
cg19377250	*SLC12A9*	−1.086	0.34	(0.1–1.1)	0.0715	0.128
cg05567269	*TJP1*	−0.912	0.40	(0.14–1.15)	0.0887	0.1507
cg22392666	*LGI4*, *FXYD7*, *CTD-2527I21.4*	1.298	3.66	(0.73–18.38)	0.115	0.1847
cg27321942	*SNORD-32A,34,33,35A*, *RPL13A*	−0.891	0.41	(0.13–1.26)	0.1195	0.1847
cg02360980	*LTK*	0.605	1.83	(0.83–4.05)	0.1354	0.2001
cg24332685	*ATP6V1G2-DDX39B*, *DDX39B*	−0.510	0.60	(0.29–1.23)	0.162	0.2294
cg05990312	.	−0.606	0.55	(0.22–1.33)	0.1816	0.247
cg04907173	*POLA2*	−0.761	0.47	(0.14–1.56)	0.2168	0.2835
cg18105612	*RP6-65G23.5*	−0.396	0.67	(0.34–1.34)	0.2611	0.317
cg26305174	*TRIP6*, *SLC12A9*	−0.694	0.50	(0.15–1.64)	0.2518	0.317
cg02675527	*PAX8*, *PAX8-AS1*	−0.460	0.63	(0.27–1.5)	0.2965	0.3476
cg18755783	*SPG20-AS1*, *SPG20*	0.202	1.22	(0.57–2.65)	0.6077	0.6888
cg21884421	*IGDCC3*	0.153	1.17	(0.55–2.48)	0.6915	0.7584
cg11567854	*NR4A1*	0.192	1.21	(0.36–4.05)	0.7549	0.7719
cg25143247	*PACRG*	0.253	1.29	(0.23–7.13)	0.7718	0.7719
cg27570661	.	−0.196	0.82	(0.22–3.09)	0.7719	0.7719

**Table A4:** Top 25 upregulated and downregulated genes in high risk patients compared to low risk patients. Abbreviations: FC = fold change; FDR = false discovery rate (Benjamini–Hochberg).

Upregulated Genes				Downregulatd Genes		
Gene	Log2 FC	FDR		Gene	Log2 FC	FDR
*CHGA*	4.35	$2.17\times 10^{-24}$		*CYP1A2*	−3.56	$2.76\times 10^{-22}$
*CA9*	3.95	$1.17\times 10^{-24}$		*CYP3A4*	−2.91	$2.30\times 10^{-18}$
*DMBT1*	3.91	$2.00\times 10^{-26}$		*C15orf43*	−2.85	$9.56\times 10^{-18}$
*UCHL1*	3.75	$1.18\times 10^{-37}$		*INS-IGF2*	−2.84	$2.23\times 10^{-11}$
*HMGA2*	3.53	$1.13\times 10^{-15}$		*SLC6A2*	−2.79	$8.92\times 10^{-08}$
*HAVCR1*	3.51	$2.76\times 10^{-22}$		*LUZP2*	−2.72	$5.82\times 10^{-14}$
*AGR2*	3.29	$2.70\times 10^{-11}$		*HAMP*	−2.64	$1.34\times 10^{-16}$
*MUC5B*	3.25	$1.90\times 10^{-14}$		*AQP6*	−2.33	$2.96\times 10^{-10}$
*CXCL5*	3.14	$3.34\times 10^{-17}$		*ECEL1*	−2.32	$7.64\times 10^{-08}$
*SLC7A10*	3.09	$1.74\times 10^{-14}$		*CRHBP*	−2.29	$8.14\times 10^{-20}$
*CLDN18*	3.06	$3.68\times 10^{-17}$		*WNT3A*	−2.28	$7.03\times 10^{-07}$
*UGT1A7*	3.05	$6.82\times 10^{-12}$		*RGSL1*	−2.26	$4.54\times 10^{-08}$
*IL20RA*	3.03	$3.14\times 10^{-13}$		*CLEC4G*	−2.19	$1.31\times 10^{-12}$
*MEP1A*	2.97	$8.12\times 10^{-16}$		*FCN2*	−2.12	$9.01\times 10^{-12}$
*WNT7B*	2.95	$7.85\times 10^{-15}$		*CYP1A1*	−2.08	$6.53\times 10^{-10}$
*CDH17*	2.84	$9.89\times 10^{-14}$		*STAB2*	−2.07	$5.00\times 10^{-11}$
*CHGB*	2.65	$4.34\times 10^{-09}$		*WBSCR17*	−2.02	$2.13\times 10^{-10}$
*ALDH3B2*	2.64	$3.55\times 10^{-12}$		*RSPO3*	−2.00	$5.77\times 10^{-10}$
*SLC34A2*	2.64	$3.15\times 10^{-10}$		*MT1G*	−1.98	$1.49\times 10^{-09}$
*MMP1*	2.63	$1.36\times 10^{-25}$		*CYP26A1*	−1.96	$3.24\times 10^{-12}$
*MMP10*	2.57	$1.34\times 10^{-14}$		*COLEC10*	−1.92	$4.44\times 10^{-15}$
*MMP12*	2.57	$7.72\times 10^{-16}$		*RSPO2*	−1.88	$1.08\times 10^{-06}$
*CHRNA1*	2.52	$2.30\times 10^{-15}$		*HEPN1*	−1.87	$4.50\times 10^{-05}$
*SCG3*	2.50	$2.17\times 10^{-14}$		*TG*	−1.85	$5.41\times 10^{-13}$
*AFP*	2.46	$1.71\times 10^{-11}$		*SAA2*	−1.84	$1.15\times 10^{-08}$
